# Supplementary material for: A Tailored Strategy to Crosslink the Aspartate Transcarbamoylase Domain of the Multienzymatic Protein CAD
Source: Molecules. 2023 Jan 9;28(2):660. doi: 10.3390/molecules28020660 (PMC9863657; doi:10.3390/molecules28020660)
Supplement: Supplementary file 1 [file molecules-28-00660-s001.zip › molecules-2067955-supplementary.pdf]

molecules

Supplementary Materials: The following supporting information can be downloaded at: www.mdpi.com/xxx/ and supplementary materials contain: Figure S1, Figure S2 and Table S1.

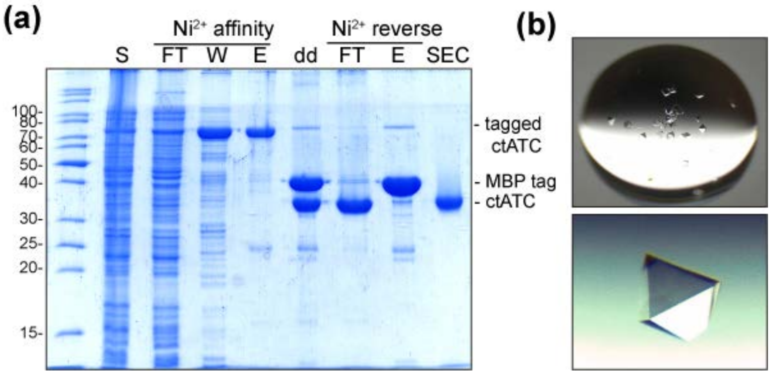

Figure S1. **ctATC2Cys purification and crystallization**. **(a)** SDS-PAGE of different steps along the purification of ctATC2Cys: S, soluble fraction of the lysate; Ni^{2+} affinity FT, W, and E, are the flowthrough, wash with 35 mM imidazole and elution of the first affinity column; dd, sample after dialysis and digestion with PreScission; Ni^{2+} reverse FT and E are the flowthrough and elution of the second affinity column; SEC, sample after size exclusion chromatography. **(b)** Bipyramidal-shaped crystals of ctATC2Cys.

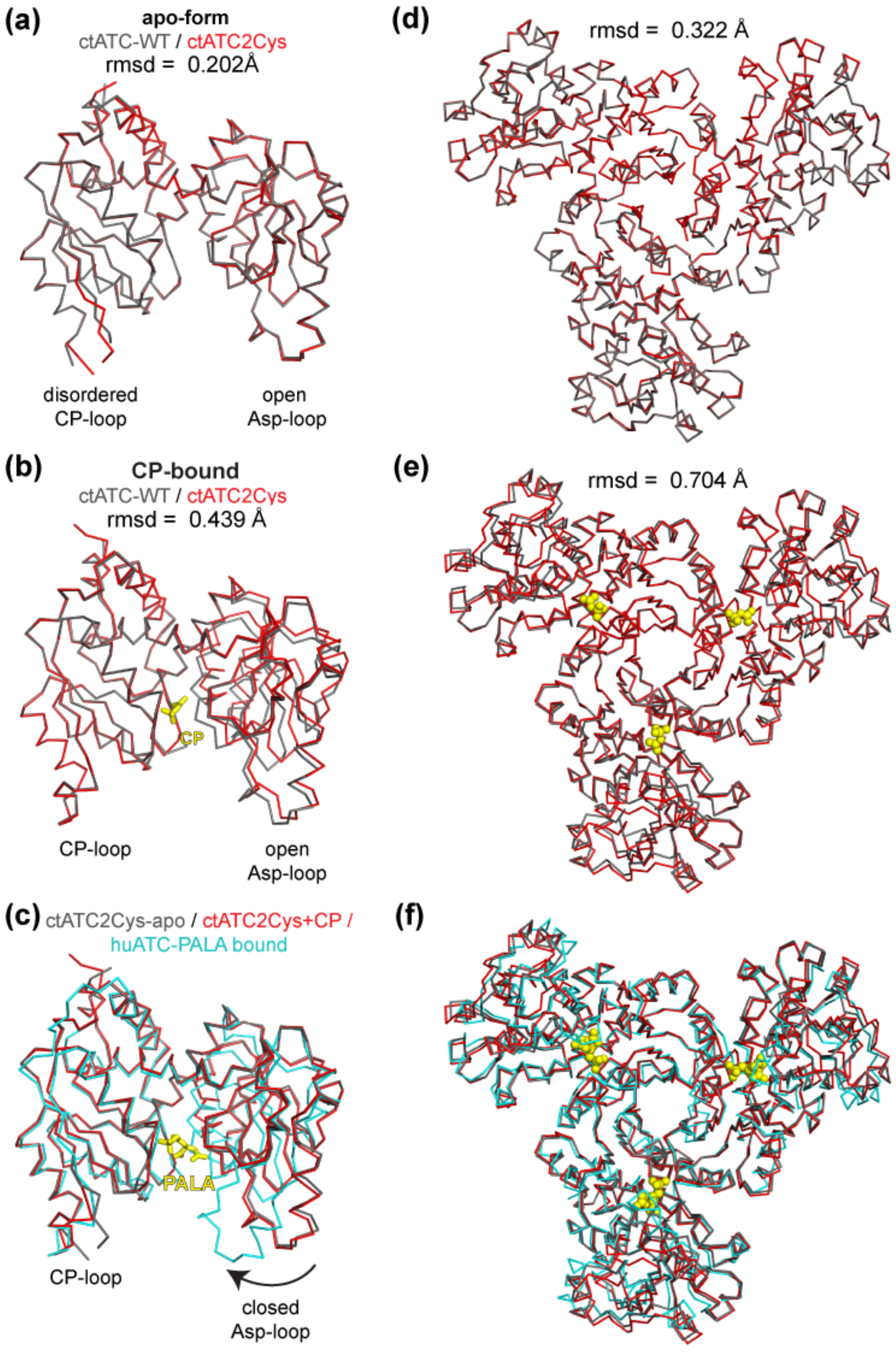

Figure S2. Structural similarity of ctATC2Cys and ctATC-WT. Ribbon superposition ctATC-WT (in grey) and ctATC2Cys (in red) subunits in apo form **(a)** or bound to CP **(b)**. **(c)** Superposition of ctATC2Cys apo and CP-bound structures with human ATC bound to PALA (shown in yellow sticks). The arrow indicates the expected movement of the Asp-loop in ctATC2Cys upon binding to PALA. **(d–f)** Superpositions for the entire protein trimer.

Table S1. Oligonucleotides used for PCR amplification and mutagenesis of ctATC. Codons for Cys mutations are in red and flanking regions for In-fusion cloning are underscored.

Oligonucleotide	Sequence 5´- 3´
ctATC-N2045C_Fw	CTG CGA ACC CTG GCC TGC TAC TCT GAT GCT ATC
ctATC-N2045C_Rv	GAT AGC ATC AGA GTA GCA GGC CAG GGT TCG CAG
ctATC-R2238C_Fw	TAT TTT CGG CAG ATG TGC TAT GGG CTG TAT TGT
ctATC-R2238C_Rv	ACA ATA CAG CCC ATA GCA CAT CTG CCG AAA ATA
ctATC-Fw	AAGTTCTGTTTCAGGGCCCTCATTCAAGAAATCTCAC
ctATC-Rv	ATGGTCTAGAAAGCTTTAGGACATGACCAGTGC
